# Efficacy of direct visual internal urethrotomy versus balloon dilation to treat recurrent urethral stricture following failed urethroplasty

**DOI:** 10.1002/bco2.458

**Published:** 2024-11-07

**Authors:** David Gilbert, Anastasia Christ, Kyle Barclay, Shubham Gupta, Kirtishri Mishra

**Affiliations:** ^1^ Case Western Reserve University School of Medicine Cleveland Ohio USA; ^2^ Urology Institute University Hospitals Cleveland Ohio USA; ^3^ MetroHealth Cleveland Medical Center Cleveland Ohio USA

## INTRODUCTION

Historically, direct visual internal urethrotomy (DVIU) and balloon dilation (BD) have been preferred as first line interventions for certain urethral strictures. Urethroplasty is considered the gold standard following failed primary intervention; however, no recommendations exist for intervention following a failed urethroplasty.^1^ Thus far, DVIU and BD have been shown to display comparable outcomes as primary treatments in terms of freedom from recurrent stricture, time to recurrence, and complications.^2^ In this research letter, we provide evidence that in the case of secondary interventions following failed urethroplasty, BD shows significantly improved 3‐year outcomes compared to DVIU.

Urethral strictures are fairly common with a prevalence of 229–627 per 100 000 males.^3^ They typically impact men over the age of 65 and increase the risk for UTIs and incontinence. While some studies have compared the success of DVIU versus BD as primary interventions, reported success rates are highly variable with 32%–96% for DVIU and 35%–84% for BD.^2^, ^4^, ^5^ Conversely, urethroplasty has a high reported success rate of 96%, though is a more complicated procedure requiring longer recovery and a skilled surgeon.^1^

Due to the low frequency of recurrence following urethroplasty, recommendations for subsequent reoperations with DVIU or BD have not been adequately studied. Given the prevalence of urethral strictures and increasing use of urethroplasty, it is important to study the success of subsequent DVIU and BD. We performed a retrospective review using TriNetX (TriNetX, Inc., Cambridge, MA, USA), a clinical research platform that collects and stores over 125 million patients' electronic health record data, to determine whether urethroplasty patients with subsequent DVIU or BD had a higher chance of recurrent stricture. We are unaware of another study that directly compares success rates of DVIU versus BD as secondary interventions following urethroplasty.

Cohorts were constructed for both DVIU following urethroplasty and BD following urethroplasty. Patient ages ranged from 21 to 90, and exclusion criteria included benign prostatic hyperplasia, neurogenic bladder and bladder neck contracture. Specific inclusion and exclusion criteria can be found in Appendix S1. Given the small sample sizes, cohorts were not matched for comorbidities. Outcomes were defined as ≥1 instance of urethral stricture or stenosis, or retention of urine between 1 month and 3 years after DVIU or BD. Outcomes were assessed with Kaplan–Meier, hazard ratios (HR) and log‐rank tests to determine significance (*p* < 0.05), and a Kaplan–Meier curve was generated.

**FIGURE 1:**
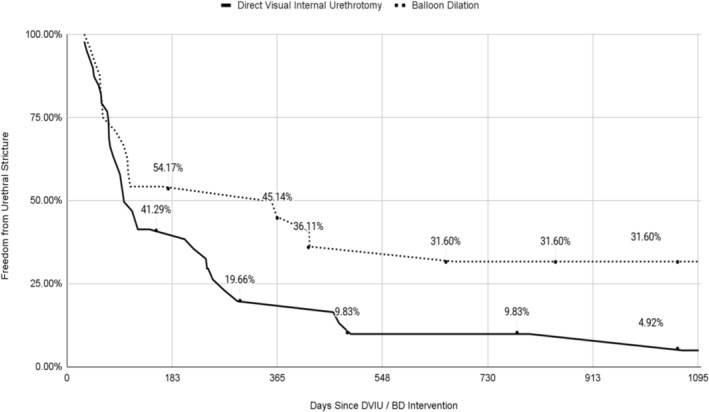
Three‐year Kaplan–Meier demonstrating probability of developing ≥1 instance of urethral stricture after secondary intervention following failed urethroplasty. DVIU/BD, direct visual internal urethrotomy/balloon dilation.

DVIU (*N* = 45) had a significantly higher probability (*p* = 0.0353) of recurrent urethral stricture compared to BD (*N* = 25), with respective 3‐year incidence probabilities of 95.15% and 69.05% (Figure 1). DVIU had a median survival of 99 days while BD had a median survival of 355 days. DVIU had an increased hazard compared to BD with a HR of 1.901 (95% CI: 1.034, 3.497). For both cohorts, the median time between initial urethroplasty and subsequent salvage intervention was comparable, with 177 days for DVIU and 153 days for BD.

In conclusion, for patients experiencing recurrent urethral stricture post‐urethroplasty, BD appears to have better 3‐year outcomes compared to DVIU. Additionally, the data suggest that in the short term, BD may provide longer lasting symptom relief before recurrence of urethral stricture.

Primary limitations of this study are attributed to the use of electronic health record data including: completeness and accuracy of medical records, loss to follow‐up and billing code restrictions. Additionally, the heterogeneity of cohorts formed through TriNetX and not a single institution's data will have significant influence and cannot be ignored—this is of particular note given the highly surgeon and hospital‐dependent outcomes of complex procedures such as urethroplasties. Lastly, due to the nature of TriNetX, we were unable to fully characterize strictures' length and location, type of urethroplasty or type of balloons used in BD. Future studies should prioritize larger sample sizes and consider a prospective randomized controlled trial to incorporate more granular data on strictures and interventional techniques, as the results of this research could change clinical management of urethral strictures.

## Supporting information


**Appendix S1.**: ICD and CPT Codes Used in Cohort Construction.

## References

[1] Benson CR , Li G , Brandes SB . Long term outcomes of one‐stage augmentation anterior urethroplasty: a systematic review and meta‐analysis. Int Braz J Urol Off J Braz Soc Urol. 2021;47(2):237–250. 10.1590/S1677-5538.IBJU.2020.0242 PMC7857757 32459452

[2] Cavalcanti AG , Fiedler G . Opinion: endoscopic urethrotomy. Int Braz J Urol Off J Braz Soc Urol. 2015;41(4):619–622. 10.1590/S1677-5538.IBJU.2015.04.03 PMC4756988 26401852

[3] Alwaal A , Blaschko SD , McAninch JW , Breyer BN . Epidemiology of urethral strictures. Transl Androl Urol. 2014;3(2):209–213. 10.3978/j.issn.2223-4683.2014.04.07 26813256 PMC4708169

[4] Beeder LA , Cook GS , Nealon SW , et al. Long‐term experience with balloon dilation for short bulbar and membranous urethral strictures: establishing a baseline in the active drug treatment era. J Clin Med. 2022;11(11):3095. 10.3390/jcm11113095 35683482 PMC9181788

[5] Kumano Y , Kawahara T , Mochizuki T , Takamoto D , Takeshima T , Kuroda S , et al. Management of urethral stricture: high‐pressure balloon dilation versus optical internal urethrotomy. Low Urin Tract Symptoms. 2019;11(2):034–037. 10.1111/luts.12208 29119701

