# Supplementary material for: Efficacy of direct visual internal urethrotomy versus balloon dilation to treat recurrent urethral stricture following failed urethroplasty
Source: BJUI Compass. 2024 Nov 7;6(1):e458. doi: 10.1002/bco2.458 (PMC11771506; doi:10.1002/bco2.458)
Supplement: Supplementary file 1 — Appendix S1.: ICD and CPT Codes Used in Cohort Construction. [file BCO2-6-e458-s001.docx]

Appendix: ICD and CPT Codes Used in Cohort Construction

| Criteria Category | Criteria Type | Description | Code | Code Type |
| --- | --- | --- | --- | --- |
| Urethral Stricture | Inclusion / outcome | Urethral Stricture | N35 | ICD-10-CM |
|  |  | Other atresia and stenosis of urethra and bladder neck | Q64.3 | ICD-10-CM |
|  |  | Postprocedural urethral stricture | N99.1 | ICD-10-CM |
| Urinary Retention | Inclusion / outcome | Other retention of urine | R33.8 | ICD-10-CM |
|  |  | Retention of urine unspecified | R33.9 | ICD-10-CM |
| BPH | Exclusion | Benign Prostatic Hyperplasia | N40 | ICD-10-CM |
| Neurogenic Bladder | Exclusion | Uninhibited neuropathic bladder, not elsewhere classified | N31 | ICD-10-CM |
| Bladder Neck Contracture | Exclusion | Bladder-neck obstruction | N32 | ICD-10-CM |
|  |  |  |  |  |
| DVIU | Inclusion | Cystourethroscopy with direct vision internal urethrotomy | 52276 | CPT |
| Balloon Dilation | Inclusion | Cystourethroscopy; with treatment of ureteral stricture (e.g., balloon dilation, laser, electrocautery, and incision) | 52341 | CPT |
|  |  | Cystourethroscopy with ureteroscopy; with treatment of ureteral stricture (e.g., balloon dilation, laser, electrocautery, and incision) | 52344 | CPT |
|  |  | Dilation of urethral stricture by passage of sound or urethral dilator, male; initial | 53600 | CPT |
|  |  | Dilation of urethral stricture by passage of sound or urethral dilator, male; subsequent | 53601 | CPT |
|  |  | Dilation of urethral stricture or vesical neck by passage of sound or urethral dilator, male, general or conduction (spinal) anesthesia | 53605 | CPT |
| Urethroplasty | Inclusion | Cystoplasty or cystourethroplasty, plastic operation on bladder and/or vesical neck (anterior Y-plasty, vesical fundus resection), any procedure, with or without wedge resection of posterior vesical neck | 51800 | CPT |
|  |  | Urethroplasty; first stage, for fistula, diverticulum, or stricture (eg Johannsen type) | 53400 | CPT |
|  |  | Urethroplasty; second stage (formation of urethra), including urinary diversion | 53405 | CPT |
